# Bacteriophages in Antarctic subglacial lakes reveal novel biodiversity and biogeochemical potential

**DOI:** 10.1093/ismeco/ycag252

**Published:** 2026-09-22

**Authors:** David M Robinson, Wei Li, Mya Breitbart, Cristina Takacs-Vesbach, John C Priscu

**Affiliations:** Department of Biology, University of New Mexico, Castetter Hall Room 1480, MSC03-2020, 219 Yale Blvd NE, Albuquerque, NM 87131-0001, United States; Physical and Life Sciences Directorate, Lawrence Livermore National Laboratory, Livermore, CA 94550, United States; Center for Advanced Microscopy and Imaging, Miami University, Oxford, OH 45056, United States; College of Marine Science, University of South Florida, Saint Petersburg, FL 33701, United States; Department of Biology, University of New Mexico, Castetter Hall Room 1480, MSC03-2020, 219 Yale Blvd NE, Albuquerque, NM 87131-0001, United States; Department of Land Resources & Environmental Sciences, Montana State University, Bozeman, MT 59717, United States; Desert Research Institute, Reno, NV 89512, United States

**Keywords:** limnology, metagenomics, virus, prokaryotes, subglacial

## Abstract

Of the more than 600 subglacial lakes identified in Antarctica thus far, Whillans Subglacial Lake and Mercer Subglacial Lake are the only lakes to have been directly and cleanly sampled. Although hydrologically isolated from direct marine contact for approximately 6.3 ± 1.0 ka, both lakes periodically discharge into the Ross Ice Shelf marine cavity. We present the first direct characterization of bacteriophages in Antarctic subglacial lakes, combining microscopy, metagenomics, and gene-sharing network analyses with bacteriophages found in the Ross Ice shelf marine cavity. The data reveal that bacteriophages are less abundant than their microbial hosts, with virus to prokaryote ratios below those of typical oligotrophic environments. Eight hundred sixty-one double-stranded DNA viral contigs were recovered forming 66 distinct viral clusters; only 18.4% could be identified at ≥85% sequence similarity in the IMG/VR database, showing high genetic novelty. The data showed 114 putative auxiliary metabolic genes involved in nutrient cycling and DNA methylation, and we predicted hosts spanning 13 prokaryotic classes. Several genetic clusters were shared with Antarctic subglacial lakes and Ross Ice Shelf cavity, indicating ecological connectivity that may extend the influence of subglacial phages to carbon and nutrient cycling in downstream Antarctic coastal ecosystems.

## Introduction

Beneath the Antarctic ice sheet lies a mosaic of subglacial aquatic habitats consisting of >600 lakes and numerous interconnected streams [1, 2]. Despite their continent-wide abundance, Whillans Subglacial Lake (SLW) and Mercer Subglacial Lake (SLM) are the only two lakes to date that have been directly and cleanly sampled through their respective 800 and 1087 m ice covers. SLW, the first subglacial lake to be sampled in January 2013, was shown to have diverse and metabolically active water column and sediment microbial communities with their metabolisms largely driven by chemolithoautotrophy and heterotrophic consumption of relict marine organic carbon [2, 3]. SLM was sampled in January 2019 and focused on water column and sediment biogeochemical carbon transformations [4]. Unlike SLW, SLM exhibited a stronger coupling between microbial activity and sedimentary carbon reservoirs, highlighting its distinct role in subglacial carbon transformation [5]. SLW and SLM are hydrologically connected, and their microorganisms appear to form a large subglacial metacommunity before eventually entering the marine cavity beneath the Ross Ice Shelf [1, 5, 6]. This hydrological outflow carries substantial organic carbon which can exceed heterotrophic microbial demand. For example, in coastal embayments along the Siple Coast, flows from inland Antarctica have been estimated to supply over 5400% of the heterotrophic carbon demand [7]. Bacteriophages (phages) may play a significant but unexplored role in mediating these carbon fluxes.

Phages are obligate intracellular parasites that are globally distributed among diverse habitats. Despite their widespread distribution, our understanding of their immense genetic diversity remains limited with many phage genes still lacking functional annotation [8]. Phages influence community dynamics and biogeochemical cycling through both lytic and temperate cycles. During the lytic cycle, phages immediately hijack host cell machinery to produce progeny, eventually resulting in the death and lysis of the host cell. During the temperate cycle, phages integrate themselves into their host genome, replicating with the host until the lytic cycle is triggered. Phages play key roles in the structure and metabolism of their host prokaryotic communities, as well as the availability of nutrients through both the viral shunt and the expression of auxiliary metabolic genes (AMGs) [9–12]. The viral shunt is predicted to release approximately 10 billion tons of carbon per day in oceanic systems through lysis alone [13]. AMGs influence host metabolism, enhancing normal metabolic functions such as biogeochemical transformations to maintain cellular activity while simultaneously supporting phage production [14–16]. These AMGs are often associated with increased metabolic rates that enable the host to accomplish both tasks [11]. While research on the role of polar phage communities has increased globally [17], little is known about phage presence or function in the subglacial Antarctic environment. Given the tremendous reservoir of carbon located under the Antarctic ice sheets [18] and the role that phages play in biogeochemical cycling in other environments, their presence in subglacial hydrological ecosystems likely has significant biological and chemical impacts under the ice and ultimately in the coastal Southern Ocean [7, 19, 20]. The first indirect evidence for the existence of phages in subglacial Antarctica was from accretion ice collected over Vostok Subglacial Lake (SLV) [21–23]. The seminal biological data from Vostok accretion ice prompted the direct sampling of sediments and the water columns of SLW and SLM [4, 22–27]. Previous studies on SLW and SLM water column and sediment samples revealed metabolically and genomically diverse prokaryotic assemblages with identified CRISPR spacers [3, 22, 24, 28]. The discovery of active prokaryotic communities in these lakes and the limited phage data from accretion ice overlying SLV motivated our study to determine phage abundance, diversity, and functional roles in the subglacial aquatic environment beneath the West Antarctic ice sheet [3, 22–24].

Here, we present the first detailed analysis of phages from the water columns of SLW and SLM. Although SLW was not sampled directly for phages, we mined previously sequenced metagenomes for phage sequences to compare with those obtained from direct sampling in SLM [3]. Sampling of SLM included a dedicated campaign to isolate and concentrate phages from water column samples. Using a combination of transmission electron microscopy, epifluorescence microscopy of DNA-stained virus-like particles (VLPs), metagenomics, and visualized gene-sharing networks we inferred the diversity, influence, and distribution of phages in these subglacial lake environments. To explore the potential larger geographical distribution of Antarctic phages, we clustered phage genomes from a permanently ice-covered surface lake in the Antarctic McMurdo Dry Valleys (MDV) and from the marine cavity beneath the Ross Ice Shelf [20, 28, 29]. Our study addressed the following hypotheses: (i) Phages in these subglacial lakes have the genetic potential to regulate the microbial abundance, diversity, and community turnover through both lytic and temperate infection strategies targeting dominant prokaryotic populations, (ii) Phages modulate host metabolic functions through the use of AMGs, reflecting oligotrophic constraints of their subglacial environment, and (iii) SLW, SLM and downstream Antarctic environments share current or past evidence of hydrologic connectivity in shared phage genetic diversity.

## Materials and methods

### Site description

**Figure 1:**
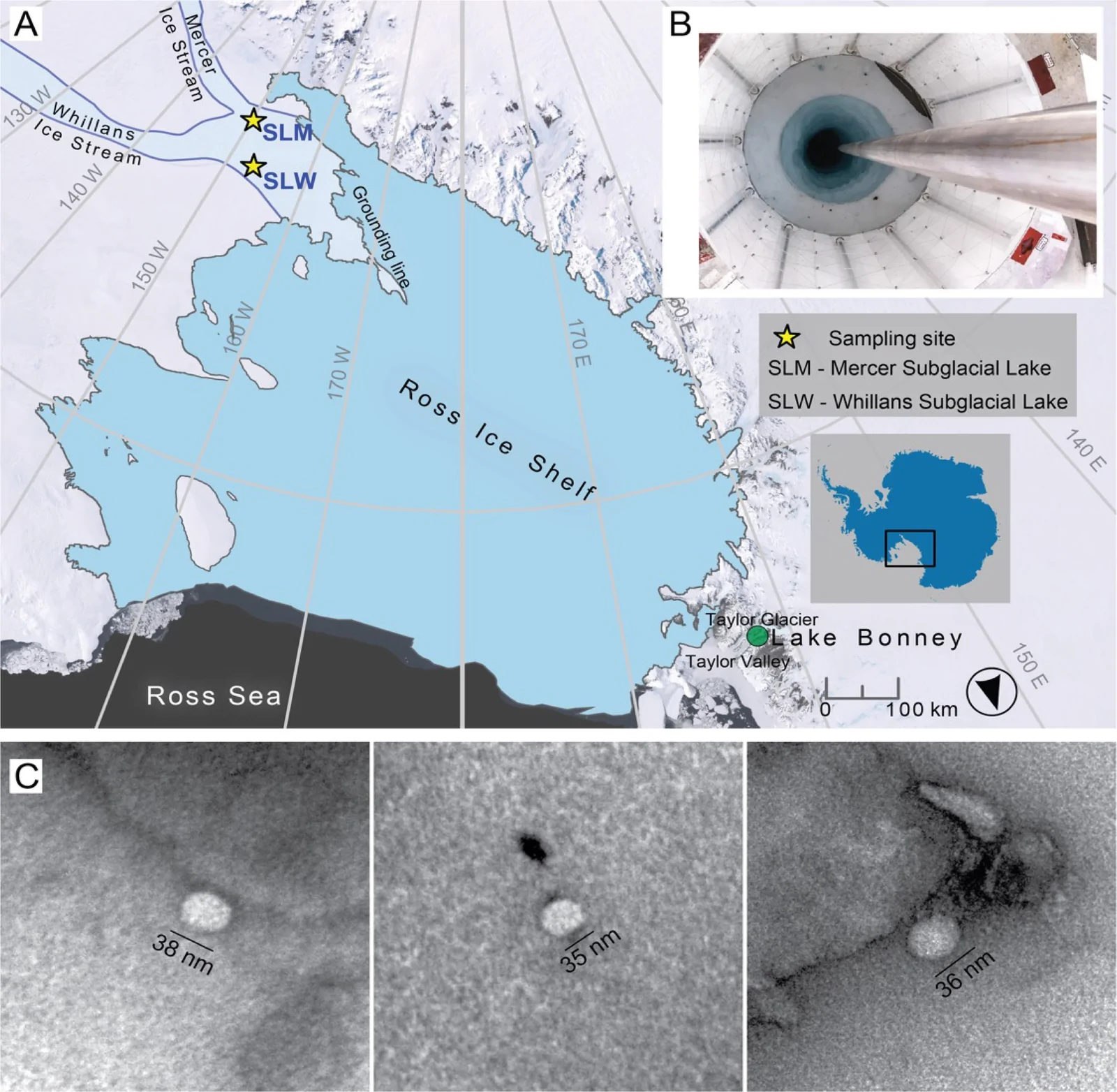
(A) Locator map of SLM and SLW relative to the Ross Ice Shelf and WLB in the MDVs. (B) View down the hot water bore drill at SLM; the white collar above the borehole contains high intensity ultraviolet light banks to sterilize all instruments deployed down the borehole (see [25] for decontamination protocols). (C) Transmission electron micrographs of VLPs observed in SLM.

SLW is located beneath the West Antarctic Ice Sheet in the lower Whillans Ice Stream under ~800 m of ice [30]. SLW has an area of ~60 km^2^ with a ~2.2 m deep water column when sampled in January of 2013. SLW undergoes approximately decadal scale drain-fill cycles; SLW was at a low stand when sampled in January of 2013 [3, 4, 31] (Fig. 1A). SLM is located beneath 1087 m of ice at the confluence of the Mercer and Whillans Ice Streams and receives ~30% of its water from East Antarctica [4, 32]. SLM has a surface area of ~143 km^2^, and a lake water column depth of ~15 m at the time of sampling; SLM was at a high stand when sampled in January of 2019 [4] (Fig. 1A). Both SLW and SLM discharge into the Ross Ice Shelf marine cavity and have been shown to fertilize the coastal environment beneath the Ross Ice Shelf adjacent to the Siple Coast [7, 32].

### Sample collection

SLW and SLM lake water was sampled via a ~0.5 m diameter borehole melted through the overlying ice. The borehole was produced and maintained using a clean hot water drilling system (Fig. 1B) employing submicron particle filtration and high-intensity UV treatment of drilling water to minimize microbial contamination, with real-time monitoring throughout drilling operations [4, 24, 25, see also Fig. 1B)]. The water samples were collected from near the middle of the water columns (1.1 and 7.5 m for SLW and SLM, respectively) close to the deepest portion of the lake using 10 l Niskin bottles precleaned with 3% H_2_O_2_ [33]. Conductivity and temperature measurements revealed no large density gradients in the water column, consistent with an environment that is well mixed vertically; hence, mid-depth samples should be representative of the water column microbial community [4, 30]. For SLM samples, subsamples were transferred cleanly to sterile vessels in surface laboratories for microscopy, phage and prokaryote enumeration, and phage sequencing. SLW samples were not collected for dedicated phage analysis and were used *post hoc* only for metagenomic mining of phage sequences as described below.

### Viral-like particle and bacterial enumerations

Triplicate lake water samples (30 ml each) from a peroxide cleaned 10 l Niskin bottle were placed in sterile 50 ml falcon tubes (Corning Inc., NY) and immediately flash frozen in liquid nitrogen at the field site. The frozen samples were transferred to a −80°C freezer before eventual shipment to Montana State University on dry ice. A total of 18 samples, including triplicates from each cast, were prepared for counting in a UV sterilized laminar flow hood at room temperature and stained with SYBR Gold (5× final concentration) (Thermo Fisher Scientific, MA) for 10 min in the dark. Five ml aliquots of each triplicate sample were filtered through 13 mm Isopore black polycarbonate membranes (0.2 μm pore size) (MilliporeSigma, MA) to collect prokaryotic cells, and the filtrates were further filtered onto 13 mm Anodisc aluminum oxide membranes (0.02 μm pore size) (Whatman, UK) to collect VLPs. Filters were mounted on glass microscope slides with ProLong® Diamond Antifade (Thermo Fisher Scientific, MA) and cells and VLPs were enumerated using a Nikon Eclipse 80i epifluorescence microscope equipped with a QIMAGING Retiga 2000R camera (QIMAGING, Canada) at an excitation wavelength of 450–490 nm and emission wavelength of 515–550 nm. Images of at least 30 random views of each sample were recorded with a 100× objective lens and the cell and VLP concentrations were calculated using ImageJ software (V1.47, National Institutes of Health, USA) [34].

### Transmission electron microscopy (TEM)

Phages for transmission electron microscopy were obtained from a single 20 ml SLM lake water sample filtered through a 25 mm Isopore clear polycarbonate membrane (0.2 μm pore size) (MilliporeSigma, MA) with a pre-combusted (450°C for 5 h) glass filter tower set (Thermo Fisher Scientific, MA). The filtrate containing free phage particles was concentrated to a volume of ~50 μl using a Nanosep Centrifugal tube (Pall, NY) with a 100 kD molecular weight cutoff (3000 g for 10 min intervals). The concentrated sample was applied to copper grids and stained with 2% uranyl acetate. Samples were viewed with a LEO912AB TEM (Carl Zeiss AG, Germany) and micrographs were captured with a Proscan charge-couple-device camera (Proscan Elektronische, Germany) [34].

### Bacteriophage DNA sequencing

Phage particles in the SLM water sample were concentrated following the protocol described in John *et al.* modified to accommodate our limited sample volume [35]. Briefly, 6 l of water sample was immediately transferred from the 10 l Niskin bottle into a 10 l TraceClean cubitainer (VWR International, PA) that had been precleaned with 1% HCl acid and triple-rinsed with sterile MilliQ water followed by triple rinsing with lake water. The sample water was then filtered through a polyethersulfone (PES) capsule filter with a 0.2 μm pore size to collect the cellular fraction. The inlet and outlet of the filter capsule was capped, then the filter was flash frozen in liquid nitrogen, and stored and transported at −80°C before DNA extraction. To obtain the phage fraction the filtrate was collected into another clean 10 l high density polyethylene collapsible container cleaned as described above and precipitated with 0.3 ml of FeCl_3_ stock solution (10 g l^−1^) to obtain the phage fraction [35], which was then filtered onto a 0.02 μm polycarbonate membrane filter and stored in a 50-ml falcon tube for transfer to the laboratory at Montana State University at 4°C in the dark. The phage particles on the filter were resuspended in 6 ml of freshly made resuspension buffer (0.1 M EDTA, 0.2 M MgCl_2_, 0.2 M ascorbic acid, pH = 6.5) and incubated on an orbital shaker at 4°C in the dark for 10 h. The supernatant was concentrated to a volume of ~200 μl using a Nanosep Centrifugal tube (Pall, NY) with a 100 kD molecular weight cutoff (3000 g for 10 min intervals). DNA was extracted from the suspension using Wizard Prep resin and mini-columns (Promega, WI) at a ratio of 0.2 ml virus suspension to 0.5 ml Wizard Prep resin. To generate enough DNA for metagenomic sequencing, a whole genome amplification step was carried out on the extracted phage DNA samples using a GenomiPhi V3 DNA amplification kit (Cytiva, MA) following manufacturer’s instruction. Amplified DNA samples were then purified with a Wizard DNA Clean-up kit (Promega, WI). DNA extraction on the cellular fraction collected on the capsule filter was performed using a DNeasy PowerSoil Pro DNA isolation kit (QIAGEN, Germany) following the manufacturer’s instructions [34]. This DNA extracted from the cellular fraction was also used for metagenomic mining of phage sequences associated with prokaryotic cells, hereafter referred to as the cellular fraction. This approach captures both integrated prophages and phages actively replicating within host cells at the time of sampling. Metagenomes from the SLW cellular fraction, previously sequenced as part of Achberger *et al.* [3], were similarly mined for phage sequences for comparative purposes. To rule out potential contamination, we included negative controls in our DNA extractions and whole-genome amplification using nuclease and nucleic acid free water in place of sample. DNA concentrations in negative controls were below background levels, confirming the absence of contaminating DNA. Sequencing was performed by BGI Genomics (CA) on an Illumina HiSeq 2500 system, generating paired-end reads of 150 bp. Sequencing produced approximately 80 million high-quality reads, corresponding to a total output of about 12 Gb (Supplemental Table S1).

### Bacteriophage assembly, discovery, QC, and annotation

The sequenced raw reads were quality controlled using the read_qc module of the metaWRAP pipeline to remove sequencing adapters and bases with quality scores below Q33 [36]. Metagenomic reads from the SLM phage fraction, SLM cellular fraction, and SLW cellular fraction were co-assembled using MEGAHIT version 1.2.9 using default parameters and a k-list of 21, 33, 55, 77, 99, 127 [37]. Putative phage contigs were identified with VirSorter2, VIBRANT, VIBRANT-Virome, Seeker, and DeepVirFinder through the What-the-Phage (v1.1) pipeline [38]. Contigs with scores higher than 0.75 from any individual tool were retained as an initial broad filter, with the intent that downstream quality control steps would further refine this set. All pooled contigs were concatenated and de-replicated through MMseqs2 easy-linclust (--min-seq-id 0.95 -c 0.8 --cov-mode 1 --cluster-mode 2) to collapse identical sequences prior to quality assessment [39]. Downstream analyses were restricted to putative viral contigs ≥5 kb in length. Quality control was performed with CheckV (v1.2.2) in the end-to-end mode to remove host contamination and estimate completion and contamination [40]. We manually inspected the remaining contigs and retained viral segments containing ≥1 viral hallmark gene (e.g. capsid, terminase, portal, tail/baseplate, packaging adenosine triphosphatease) and no bacterial single-copy or ribosomal genes (contigs containing at least one viral hallmark gene and no more than two predicted host genes). Contigs that passed quality control steps were re-assessed with Virsorter2 to remove single stranded DNA (ssDNA) viruses associated with phi29 polymerase amplification bias during library preparation and were prepared for AMG annotation by DRAM-v by generating a phage-affiliated contigs table [41, 42]. This multi-step quality control framework ensured that only high-confidence viral contigs were retained for downstream analyses. Final phage contigs from the co-assembly of all three metagenomes were clustered at the genus level (gene sharing networks based on protein clusters) in vConTACT2 v.5 using the NCBI RefSeq Release 201 [34]. To assess the accuracy of the final phage contig set, we randomly selected forty viral contigs and blasted them to search for hallmark regions and host contamination.

### Annotation of auxiliary metabolic genes, host predictions, lifestyle and quantification

AMGs were annotated from the final phage assembly using DRAM-v v1.3.2 [42], which annotates genes against multiple databases including KEGG, Pfam, VOGDB, MEROPS, and dbCAN providing carbohydrate-active enzyme classification based on the CAZy database [43]. Based on the output of DRAM-v, only high confidence AMGs in auxiliary score categories 1–3 were retained, corresponding to genes with strong evidence of viral origin and metabolic function, with no flags indicating transposase activity or location at contig edges. AMGs were additionally confirmed by visual inspection of gene neighborhood context. Glycosyltransferases identified through dbCAN annotations were retained following manual inspection of gene neighborhood context. These genes received MKF flags in DRAM-v, indicating they are metabolic genes with prior AMG literature support, located near contig ends. DNA cytosine methyltransferases (*dcm*) received M and F flags; while these lack the K flag indicating prior AMG confirmation, their retention is supported by their consistent recovery in previous studies [44, 45]. To further validate the origin of the ConA-like lectin/arabinase, glycosyltransferase (GT), and *dcm* gene families, we used maximum-likelihood phylogenetic trees, constructed for each gene set. Protein sequences were aligned with their top BLASTp hits (NCBI nr, e-value <1e-5) using MAFFT, and trees were built with FastTree under default parameters and visualized in iTOL (Supplementary Figs. S2–S4) [46, 47]. Potential microbial hosts were predicted using iPHoP (Integrated Phage Host Prediction) against the iPHoP August 2023 reference database, which incorporates prokaryotic genomes from NCBI RefSeq, IMG, and GTDB [34, 48]. We predicted hosts for our phage contigs, retaining quality scores of 0.90 and higher as determined through iPHoP. In total we retained 78 predictions, which was approximately 9.05% of the total contigs. Phage lifestyles were predicted by using PhaTYP, predictions assigned 0.90 and higher were retained [49]. Coverage values were generated by mapping the quality-controlled reads to the curated double-stranded DNA (dsDNA) final contigs using minimap2 (v2.28) with the -ax sr flag and secondary alignments were disabled (--secondary = no) [50]. Alignments were sorted and indexed with samtools (v1.19) [51]. Read coverage and abundance were quantified directly from BAM files using CoverM (v0.7.0) in *contig* mode with stringent filters including, --min-read-percent-identity 95 --min-read-aligned-percent 80 and --min-covered-fraction 0.75 [52]. Relative abundance was reported as transcripts per million, as implemented in CoverM; length-normalized coverage per million reads. Phage contigs were considered present in a sample when genome breadth, defined as the proportion of contig positions covered by at least one read, was ≥75%, following established thresholds for viral metagenomics [53–55]. These abundances were used for all quantifications.

### Bacteriophage taxonomy assignment, visualization, and sequence novelty assessment

Phage taxonomic annotation of SLW and SLM phage datasets was performed with vConTACT2 (v.5) using the NCBI RefSeq Release 201 viral protein database and the DIAMOND protein alignment tool [56, 57]. To determine phage distribution across Antarctic environments, we generated a broader gene-sharing network incorporating phage contigs from SLW and SLM alongside publicly available datasets from Lake Bonney in the McMurdo Dry Valleys [20, 58] and the Ross Ice Shelf marine cavity [29]. Phage contigs from these external datasets were obtained from their respective publications and combined with the SLW and SLM contigs for network analysis. Protein-coding genes predicted from viral contigs identified by VirSorter2 were clustered together in vConTACT2 (v.5) following the parameters described in Guo *et al.* [41, 59] and visualized in Cytoscape v3.8.0 using an edge-weighted spring-embedded model [60]. Viral contigs were compared against the IMG/VR v4.1 database using BLASTn [61]. Searches were run with an e-value cutoff of 1e-5, a minimum percent identity of 70%, a minimum alignment length of 500 bp, and restricted to the top scoring hit per query.

## Results and discussion

### Low virus to prokaryote ratios reflect oligotrophic conditions and diverse infection strategies

TEM analysis revealed abundant spherical VLPs that were generally smaller than 50 nm in diameter (Fig. 1C). While morphology alone cannot resolve viral taxonomy, the observed particle size distribution agrees with previous reports showing that small viruses dominate freshwater and other aquatic viral communities [62, 63]. Epifluorescence microscopy showed VLP abundances in SLM between 1.2 × 10^4^ ml^−1^ and 1.9 × 10^4^ ml^−1^ (Table 1). The abundance of VLPs in SLM is lower than most temperate aquatic locations and other environments around Antarctica [34]. For example, in the surface lakes of the MDV region of Antarctica, VLP abundances vary between 0.47 × 10^6^ ml^−1^ and 45.48 × 10^6^ ml^−1^, which are higher than their prokaryotic counterparts and are similar to VLP abundances in some temperate locations such as ground fed springs or Yellowstone National Park thermal springs [64–66]. Prokaryotic cell counts in SLM vary between 1.33 × 10^4^ ml^−1^ and 2.54 × 10^4^ ml^−1^ across sampling casts, which are also low compared to temperate and other Antarctic locations [64]. The virus to prokaryote ratios (VPRs) in SLM vary between 0.47 and 0.75 which are lower than most reported VPR values in oligotrophic environments [67–69] (Table 1). Because phage productivity depends on the growth and turnover rates of host cells, we expected the low energy availability (e.g. no sunlight) in subglacial environments to decrease VPRs. Prokaryotic cells in subglacial lakes are affected by several biotic (organic sources and lack of grazing) and abiotic factors (temperature, pressure, darkness), distinct from lakes that support photosynthesis, which may affect phage dynamics and abundances. However, despite low VPRs, genomic evidence indicates that subglacial lake phages have diverse infection strategies, including both lytic and lysogenic (temperate) lifestyles. Lifestyle predictions from metagenomic sequences should be interpreted with caution, particularly for shorter contigs. To mitigate this, we restricted analyses to contigs ≥5 kb and retained only high confidence PhaTYP predictions (≥0.90), which reduces but does not eliminate uncertainty associated with fragmented genomic sequences. Hydrologic pulses in subglacial lakes may increase host productivity and intermittently favor lytic infections whereas dormant periods may favor largely lysogenic or inactive phages which is consistent with other low energy systems [70].

**Table 1:** Table showing epifluorescence microscopy counts for VLP∙ml^−1^, prokaryotic cells (Cells∙ml^−1^), and VPR from specific water column sample collections (Casts) in Lake Mercer. It is likely that the sample bottle in cast two was triggered early, diluting the sample with borehole glacial meltwater.

Cast	VLP∙ml^−1^	Cells∙ml^−1^	VPR
1	14 485.1	23 914.5	0.605
2	3049.5	13 321.5	0.228
3	12 037.5	25 399.1	0.473
4	19 300.1	23 914.5	0.807
5	14 806.1	20 330.0	0.728
6	17 133.4	22 844.5	0.75

VLP, viral-like particles; VPR, virus to bacteria ratios.

### Subglacial Lakes exhibit unique bacteriophage communities

**Figure 2:**
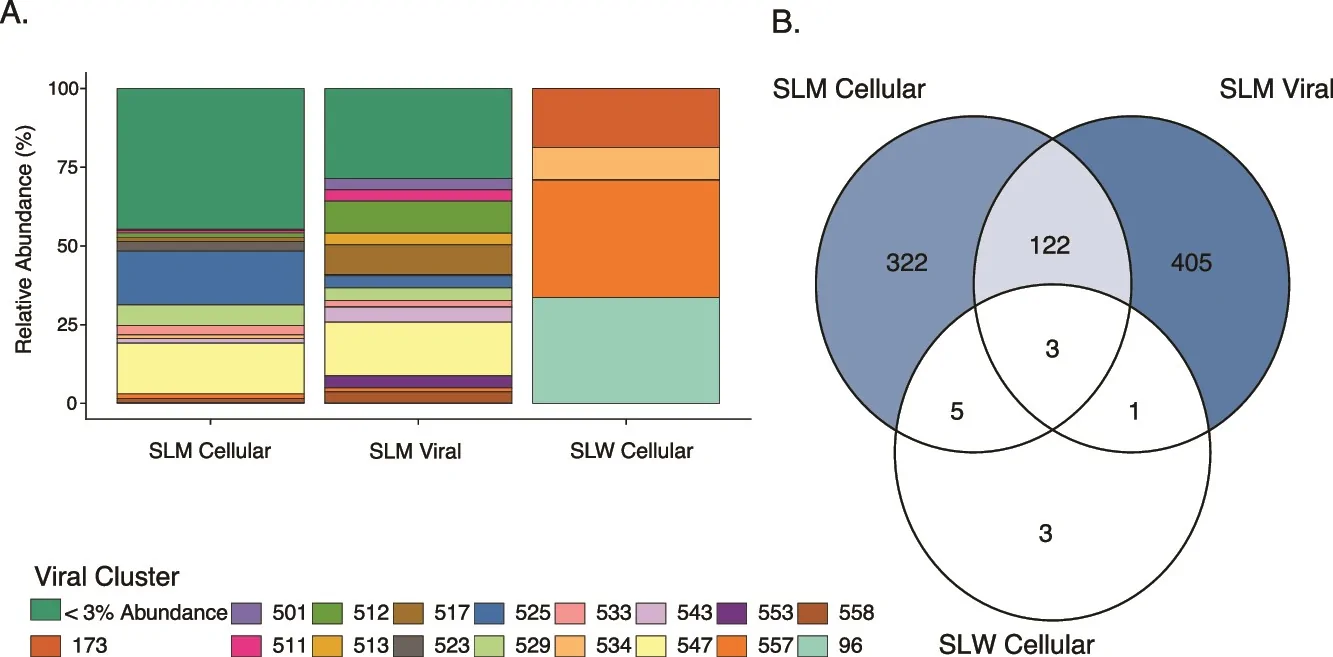
(A) Relative abundance of VCs assigned by vConTACT2 comprising greater than 3% of the total community in each fraction. VC identifiers are shown in the legend; unlabeled clusters each contributed less than 3% of relative abundance and are grouped as “<3% Abundance.” The lower number of VCs recovered from SLW cellular reflects comparatively lower sequencing depth of the SLW metagenomes. (B) Venn diagram showing the number of shared and unique phage contigs across the three fractions.

Planktonic and cellular phages were sequenced separately in SLM, whereas in SLW, we exclusively used the cellular metagenomes collected on a 0.2 μm filter because no planktonic (cell-free) phage fraction was collected. Our metagenomic analysis for all samples is based on 861 unique double stranded DNA phage contigs greater than 5 kb in length. Classification of phages in environmental samples is inherently difficult due to factors such as the lack of universal marker genes in phage (c.f., ribosomal RNA genes in prokaryotes and eukaryotes), the limited number of phage reference genomes, and high genetic variability [41, 56]. vConTACT2 clustering resulted in phage clusters (VCs) that represent putative phage genera. Of these 66 clusters, only one of them was classified within the RefSeq201 database. To further validate the novelty of these viral contigs we compared them against all contigs available in the IMG/VR v4.1 database using BLASTn. Of the 861 contigs, 159 (18.4%) contigs had sequence similarity of 85% and above. The low percentage of identification is consistent with other Antarctic studies that use similar methods. For example, in a recent study of Antarctic endolithic communities, only 1.8% (41 out of 2286) of total VCs were able to be identified by relevant databases [71]. Based on sequence similarity, subglacial lake viruses likely represent novel taxa across all taxonomic groups. We also paired the read-mapped abundances of the mined phage contigs to their VC groupings and plotted the major VCs contributing to over 3% of the total sample (Fig. 2A). Many of the most abundant VCs were unique to each fraction except for the SLM cellular and phage fraction, which shared nine major clusters (Fig. 2B).

Phage diversity in Antarctica surface aquatic environments is notably high, especially when compared to other ecosystems across our planet. For example, using similar metagenomic classification methods in MDV Lakes Bonney and Fryxell, 856 and 1360 phage clusters were reported, respectively [20]. These authors partially attributed this trend to the microbially dominated ecosystem and lack of a complex food web, which enable phage to have more specialized roles leading to greater speciation and diversity. However, in SLW and SLM this level of diversity was not observed despite the similarly truncated food chains of these subglacial systems [64, 72]. Differences in diversity between Antarctic surface and subglacial lake environments cannot be attributed to sequencing coverage alone as the 16S rRNA gene sequences also denote lower diversity in subglacial lakes relative to other surface locations around Antarctica [3, 24, 73]. Hydrological dynamics likely contribute to this disparity. MDV surface lakes are perennially ice-covered and characterized by minimal flushing and negligible water turnover. On the other hand, subglacial lakes undergo periodic drain-fill cycles on decadal timescales which effectively resets the water column community. Phage diversity could be controlled by the oligotrophic conditions present in the subglacial environment in a manner like the prokaryotic community. The lower prokaryotic diversity, lower bacterial production and relatively high hydraulic turnover (the lakes fill and drain on ~decadal scales) in oligotrophic subglacial lake systems likely also limit host-phage interactions, reducing viral lysis and suppressing microbial productivity and functional diversity. Additionally, the lower metabolic rates of microbial communities in these environments could reduce the expression of genes involved in phage-host interactions, such as those coding for phage receptors, potentially constraining viral infection and lysis.

### Shared bacteriophage distribution across Antarctica

To assess phage community distribution in SLW and SLM, we compared all phage contigs, including ssDNA phages, instead of only the phage clusters generated from vConTACT2 allowing us to include singletons for a more extensive analysis. Phage partitioning among the subglacial lakes was highly segregated, with SLW and SLM sharing approximately 1% of the total dsDNA phage contigs. When compared with the total number of all contigs, the SLM phage fraction had the highest percentage of unique contigs (47%) followed by the SLM cellular fraction (37%), and SLW (0.34%) cellular fractions. The SLM phage and cellular fractions shared the most contigs (14.5%), while the SLM cellular fraction shared the most contigs with the SLW cellular fraction (0.9%) rather than the phage fraction from SLM (Fig. 2B). Unique phage contigs did occur in the cellular fraction of SLM that were not detected in the free phage fraction, revealing characters of a temperate viral lifestyle strategy. The differences between the contig distributions between SLM and SLW presumably reflect the differences in the physical, chemical, and biological properties between SLW and SLM. SLW and SLM are ~60 km apart and thought to be connected by upstream hydrology (Fig. 1A), which is reflected in the presence of shared phage contigs [5, 31].

**Figure 3:**
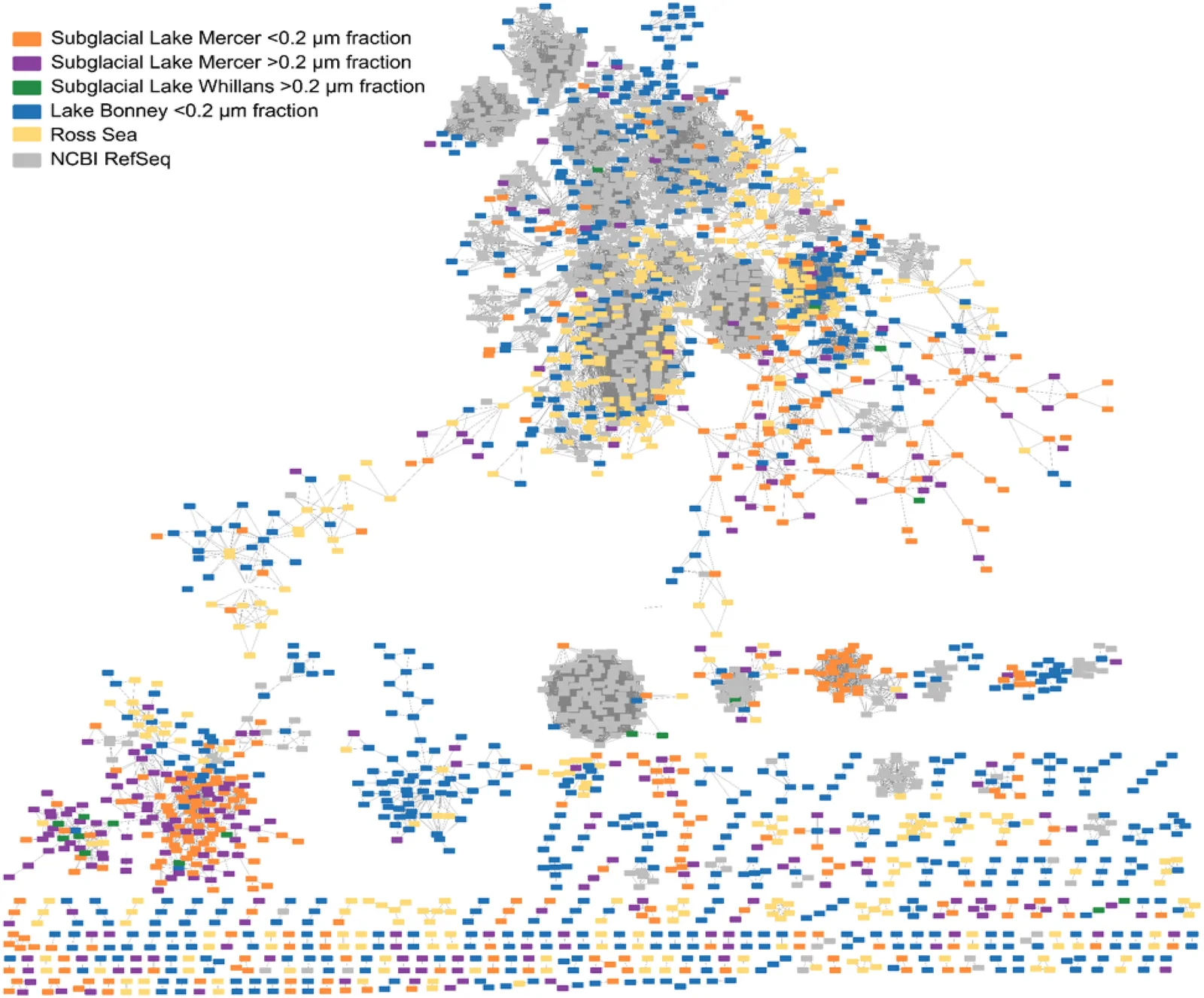
Cytoscape bacteriophage genome cluster network shows viral contigs clustered by sequence similarity, with nodes representing contigs and edges denoting shared sequence features. Clusters of contigs show patterns of shared sequence similarity, with notable overlaps between Antarctic environments, indicating genetic connections between Antarctic ecosystems. The relatively low overlap between our samples and the NCBI RefSeq data (denser clusters of grey) highlight the uniqueness of the Antarctic community relative to the database.

To expand our analysis on phage distribution, all the mined subglacial lake phage contigs, including ssDNA phages were clustered with the RefSeq201 database. Although ssDNA phage contigs were excluded from quantitative analyses due to phi29 amplification bias, they were retained for the qualitative vConTACT2 network which is based on shared protein content rather than abundance. Notably, our sequences did not cluster with the database so additional *Microviridae* genomes were added, as well as phage contigs identified from a permanently ice-covered (~4 m) Antarctic surface lake in the MDV (east lobe Lake Bonney, 1826 contigs), and phage contigs from a survey in the Ross Sea (639 contigs) [29]. The additional *Microviridae* genomes were included to cluster with potential ssDNA viruses. The network generated through vConTACT2 was visualized with Cytoscape v3.8.0 (Fig. 3). To determine genetic overlap in the maximum number of phages in different environments, phage contigs were mined from single assemblies and we included single stranded viruses. In total we identified 196 phage clusters among SLM cell and phage fractions, SLW, Lake Bonney, and the Ross Sea curated viromes. One of the phage clusters was unique to SLW while more than half (56%, *n* = 110) were found only in SLM samples. The lower number of viral clusters recovered from SLW (12) likely reflects the comparatively lower sequencing depth of the SLW metagenomes, which were generated from earlier sampling campaigns. Given the extraordinary logistical difficulty of accessing these environments, we include the SLW dataset as valuable complementary data noting its limitations. Four phage clusters were formed solely by subglacial lake samples (SLW and SLM). Another significant portion (34%, *n* = 66) of the subglacial samples clustered exclusively with the Lake Bonney and Ross Sea libraries, highlighting the unique selection pressure and isolation of the Antarctic environments. The unique clustering was also visualized in the global phage network plot (Fig. 3).

### Phages encode putative auxiliary metabolic genes reflecting subglacial biogeochemical constraints

**Figure 4:**
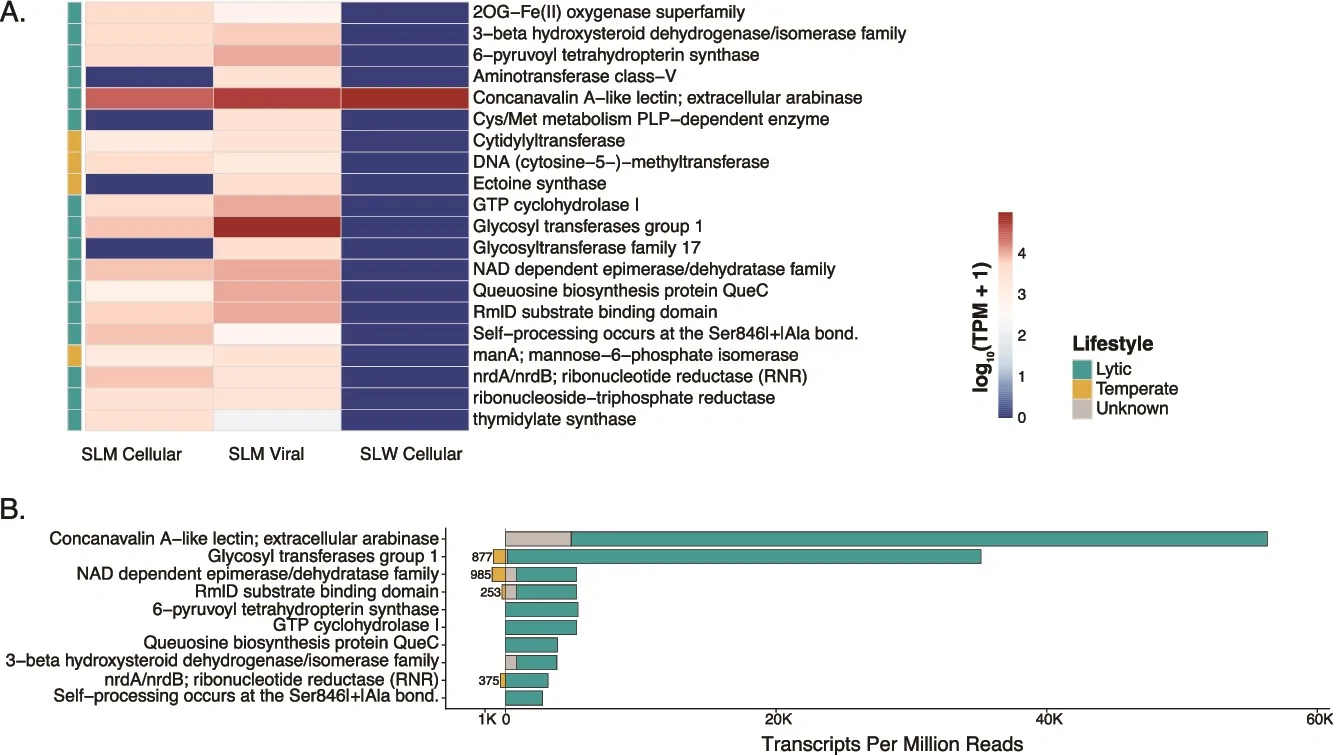
(A) Heatmap of the top 20 most abundant putative AMGs identified in Lakes Whillans and Mercer. AMGs were identified with DRAM-v and abundances were quantified as TPM, calculated as length-normalized coverage per million mapped reads. Heatmap values represent log₁₀(TPM + 1) transformed abundances. Row annotations indicate the dominant predicted lifestyle (Lytic, Temperate, or Unknown) of the phage contig encoding each AMG. (B) Mean TPM of the top 10 AMGs across all samples, stacked by predicted phage lifestyle (Lytic, Temperate, or Unknown). TPM, transcripts per million.

Phages encode AMGs that have the potential to modulate host metabolism and confer adaptations to their environment, though caution is necessary as not all predicted AMGs are currently verified metabolically active genes [11, 12, 74]. We identified 114 putative AMGs in the subglacial lake phage contigs, though 96 (84%) lacked complete metabolic pathway classification in the DRAM-v genome summary output and were instead annotated based on gene descriptions from the underlying database hits. As such, we focused on the gene description rather than the overall metabolic classifications and paired them with their abundances and viral lifestyle to ascertain AMG strategies in the subglacial lakes (Fig. 4A). The most abundant AMG in our dataset is a “Concanavalin A−like lectin; extracellular arabinase” gene and is most associated with lytic phages across all three subglacial lakes (Fig. 4B). ConA-like lectins potentially bind specific carbohydrates within exopolymeric substances (EPS), whereas arabinase domains hydrolyze polysaccharides that contain arabinose [75]. Subglacial lake microbial life frequently occurs in EPS matrices on particles or sediment [5]. EPS provide structural support and cryoprotection but also help retain carbon and other complex polymers which can be recycled within the matrix [76]. Phages encoding this ConA-like lectin with an extracellular arabinase domain may be capable of both binding and enzymatically degrading EPS, enabling them to better breach biofilm barriers upon exit from their microbial hosts, allowing them to spread more easily within the biofilm. In these carbon limited systems, phages may accelerate short-term carbon turnover and microbial community restructuring [54, 77]. This type of phage driven control on microbial dynamics has been noted in alpine biofilms and subfreezing Arctic peat soils [54, 77]. Phylogenetic analysis placed the recovered phage sequences within a single clade distinct from surrounding bacterial diversity which supports the viral origin of this gene. This clade was nested near Bacteroidota- and Chitinophagaceae-affiliated sequences which are taxa commonly associated with polysaccharide degradation, consistent with the proposed role of this AMG in EPS degradation (Supplementary Fig. S2). Collectively, these results are consistent with this AMG being involved in EPS interaction. However, whether this is through direct metabolic degradation, structural cell-surface recognition, or biofilm penetration, we cannot tell from the metagenomic sequences alone, but this does suggest that phages may contribute to biogeochemical cycling in these cold oligotrophic environments.

The GTs in this dataset were identified from the CAZy database and are primarily involved in carbohydrate metabolism and therefore carbon manipulation [43]. Phage-encoded GTs have been reported as AMGs in diverse environments including permafrost soils, marine systems, and cyanophage genomes, where they are associated with carbohydrate metabolism and host metabolic manipulation [55, 78]. While some GT annotations may reflect viral structural roles rather than direct host augmentation, GTs have been recovered consistently across independent viromics studies [79]. Notably, we identified GTs from groups 1, 2, and 17. Many lytic viruses have been documented to use GT1 for self-modification of viral structures which can protect the phage genome from restriction enzymes or CRISPR defenses [80, 81]. GT1 may increase lytic infection by enhancing infectivity and replication success by glycosylating tail fibers proteins to improve adsorption and resist host defenses. In subglacial lakes modified glycans or receptor binding proteins could allow phages to persist longer or even maintain infectivity even across dormancy intervals. In a temperate context, phages could increase host cell fitness by enhancing stress tolerance or biofilm formation which provides advantages in cold environments. Additionally, since the prokaryotic communities in subglacial lakes are slow growing, manipulating host metabolism to increase carbon rich molecules would provide competitive advantage for both bacteria and phages. Collectively, the proposed roles for these GTs can span both structural functions, such as tail fiber modification and host recognition, and metabolic functions, such as manipulation of host carbon pools. While the specific function of these GTs remains unresolved, their phylogenetic placement and consistent recovery across independent viromics studies support their identity as genuine, phage-encoded genes worth further investigation.

Ribonucleotide reductase (RNR; nrdA/nrdB) and ribonucleoside-triphosphate reductase were recovered from five independent phage contigs in this dataset. RNR is well described as having roles in nucleotide metabolism reported in T-4 like and marine cyanophages [82, 83]. RNR catalyzes the reduction of ribonucleotides to deoxyribonucleotides which directly supplies the dNTP pool required for phage genome replication during infection [84, 85]. In nutrient limited environments such as subglacial lakes, host cell nucleotide pools may already be limited due to the oligotrophic constraints of the subglacial lake environment. RNR could allow phages to supplement or bypass this constraint which would support more efficient genome replication independent of nucleotide availability.

There are multiple predictions of DNA (cytosine-5-)-methyltransferase (*dcm*) exclusive to temperate phages. While *dcm* annotations warrant scrutiny as potential defense genes rather than canonical AMGs, phage-encoded *dcm* has been reported as the most abundantly distributed AMG in marine viromics studies, where orphan methyltransferases protect phage DNA from host restriction-modification systems [44, 79]. Consistent with this, the most defensible role for these methyltransferases is likely restriction-modification evasion upon host cell entry, which may not represent a canonical AMG function in the strict sense [34, 44, 86, 87]. However, bacterial productivity in SLM and SLW have been shown to be co-limited by N and P; catabolizing methionine could help circumvent these nitrogen deficiencies which is also a speculative role that *dcm* could fill for phages [2]. Methionine is a hydrophobic amino acid which could be important for phage capsid stability, allowing for longer persistence in the environment between infections [34, 88]. This may increase host fitness by recycling limiting nutrients but could also prevent infections from similar but lytic phages.

### Phages are predicted to infect previously reported taxa with multiple lifestyle strategies

**Figure 5:**
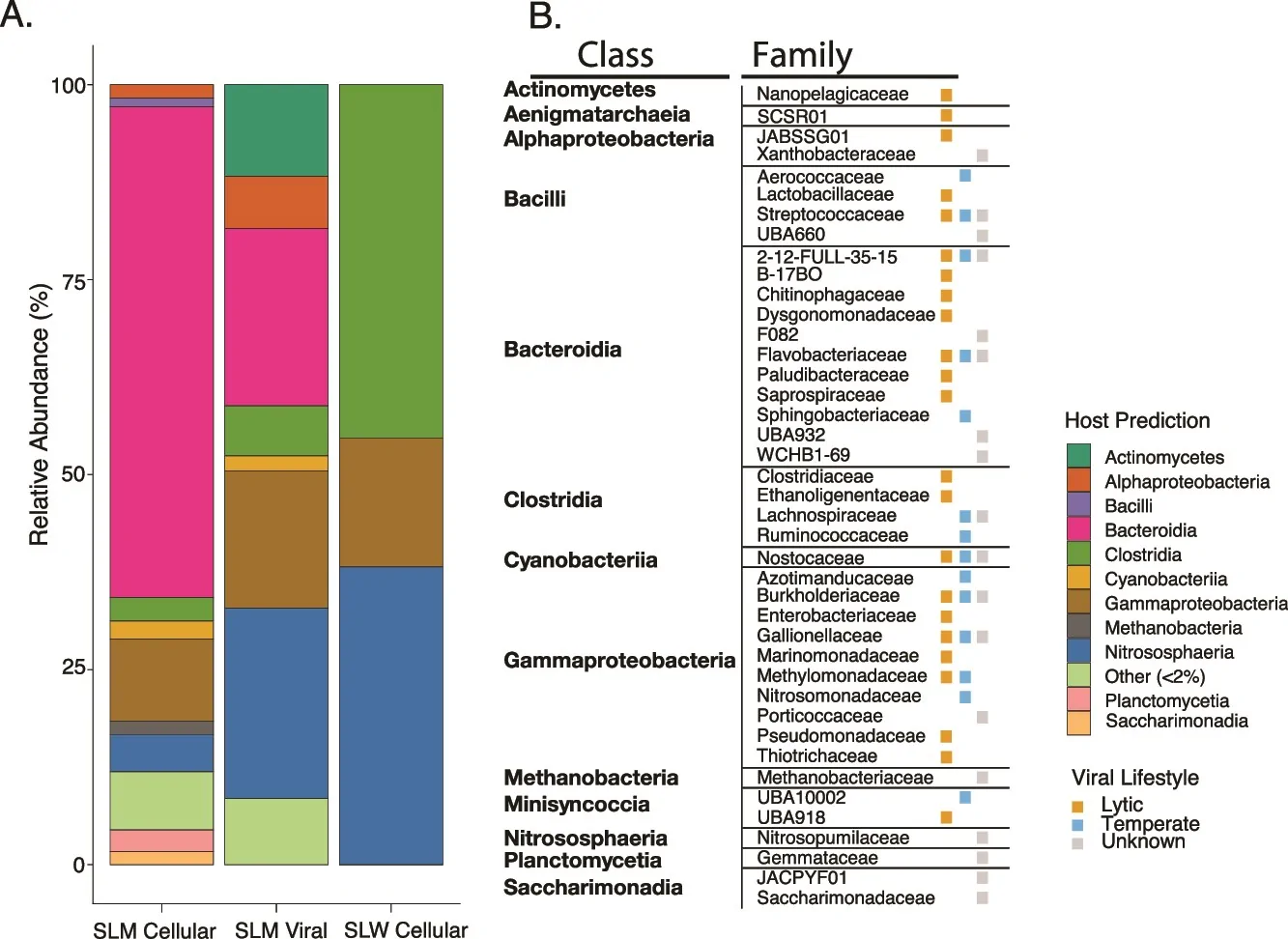
(A) Relative abundance of bacteriophage hosts predicted through iPHoP. Host predictions were paired with their corresponding bacteriophage contig abundances. (B) Prokaryotic classes and corresponding families with their predicted phage lifestyle.

Phage-host interactions have been shown to significantly influence host diversity by exerting selective pressure on bacterial populations [11, 12]. Studies of SLW and SLM have revealed a complete lack of metazoans, which increases the relative ecological and biogeochemical roles of phages in these truncated food webs [64, 89]. Phage-host pairings predicted by iPHoP revealed that phages in SLM and SLW potentially infect 13 different classes of prokaryotes. The majority of host predictions were assigned to six main bacterial classes, Bacteroidia, Gammaproteobacteria, Actinomycetes, Clostridia, Nitrososphaeria, and Cyanobacteria. To visualize relative abundances of different phage-host preferences, we paired the predicted hosts with the abundances of the corresponding phage contigs (Fig. 5A). Many of the predicted microbial hosts are previously described in other subglacial lake studies [28, 34, 90, 91]. Predicted dominant phage hosts (above 3% relative abundance) shared by all three samples include Actinomycetes, Nitrososphaeria, and Gammaproteobacteria (Fig. 5A). The dominant hosts shared between the two Mercer fractions are Bacteroidia and Nitrososphaeria. We predicted phage lifestyle to the family level (41 families) for the phage contigs assigned to the hosts and found that 16 families had exclusively lytic phages while only 7 were exclusively temperate, 11 were unknown, and 7 contained a mixture of lytic, temperate, and unknown lifestyles (Fig. 5B).

Our host predictions are similar to previously reported taxa within subglacial lakes. The presence of Cyanobacteria is intriguing, while not previously detected in Lakes Mercer or Whillans, they have been reported in the accretion ice above Lake Vostok [92]. Cyanobacterial genetic material within these phage contigs may represent relict sequences persisting from past marine incursions into the subglacial environment, consistent with evidence of ancient seawater-derived genetic material preserved in bacterial genomes from these lakes [28]. The host predictions also include potentially obligate anaerobic bacteria such as Methanobacteria and Clostridia, implying that viruses may play roles in fermentation or methanogenesis beneath the ice. Collectively, the host predictions underscore how subglacial viral community hosts are not only taxonomically diverse but are also structured in ways that highlight metabolic diversity and potential ecosystem history. We note that host predictions would be further refined by incorporating metagenome-assembled genomes (MAGs) recovered from the cellular fraction of these samples into the iPHoP reference database.

The coexistence of both lytic and temperate viral lifestyles amongst the predicted hosts suggests that mixed infection lifestyles may serve as a survival strategy in subglacial environments. Lytic infections can promote episodic carbon and nutrient turnover during transient meltwater pulses or fluxes in nutrients. Temperate phages could allow phage genomes to persist in dormant or slow growing hosts during extended periods of low nutrients and therefore low metabolic activity. Dual strategies potentially stabilize phage populations beneath the ice and contribute to maintenance of metabolic potential, genetic exchange, and ecological resilience of phage communities.

## Conclusion

Our hypotheses posed in the introduction were tested using microscopy, VLP counts, and metagenomic sequencing, to reveal the presence of phages in Antarctic subglacial lake ecosystems and indicates varying phage strategies to persist in subglacial lakes. Additionally, phages in SLW and SLM share genetic similarity with phages outside of subglacial lake ecosystems, particularly with the Ross Sea phages, indicating connectivity between environments. Collectively, these results show that phages play integral roles in subglacial carbon and nutrient mobilization and ultimate transport of nutrients to the Ross Ice Shelf cavity. Given that these subglacial lake sedimentary basins contain an estimated 21 000 petagrams of organic carbon, which is more than 10 times the carbon in Arctic permafrost, viral mediated transformation of nutrients leaving subglacial lakes could have substantial impacts on coastal Antarctic environments [93, 94]. Specifically, viral lysis releases cellular carbon and nutrients into subglacial waters that ultimately discharge into coastal Antarctic environments, indicating that phage mediated nutrient transformations may represent an underappreciated portion of this carbon flux. The degree to which phage activity shapes the compositional and bioavailability of exported nutrients warrants further investigation. Our findings underscore the importance of subglacial Antarctica as a significant source of carbon in this region, with viruses playing a central role in its transformation.

## Classification

Biological Sciences/Microbiology.

## Supplementary Material

Supplementary_material_ycag252

## Data Availability

All data generated or analyzed during this study are included in this article and its Supplemental Information files. Sequence data are available in the NCBI under accession PRJNA123222 and R code used in this analysis are available at https://github.com/Drob84761/lake-mercer-viruses
