# Supplementary material for: Bacteriophages in Antarctic subglacial lakes reveal novel biodiversity and biogeochemical potential
Source: ISME Commun. 2026 Sep 22;6(1):ycag252. doi: 10.1093/ismeco/ycag252 (PMC13615525; doi:10.1093/ismeco/ycag252)
Supplement: Supplementary file 1 [file supplementary_material_ycag252.zip › ConA_Supp2_ycag252.pdf]

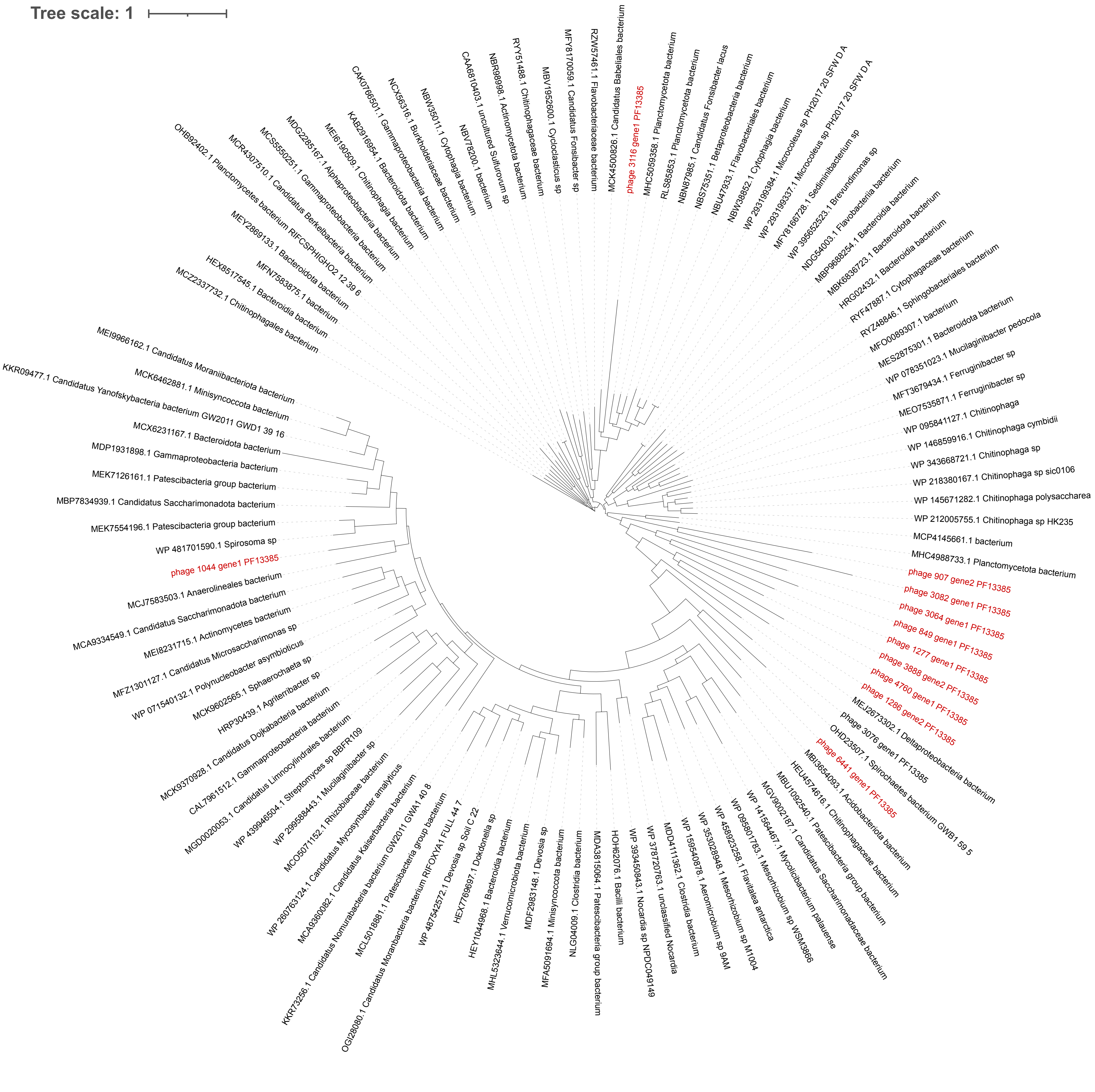
Tree scale: 1
MEI9966162.1 Candidatus Moraniibacteriota bacterium
MCK6462881.1 Minisyncoccota bacterium
KKR09477.1 Candidatus Yanofskybacteria bacterium GW2011 GWD1 39 16
MCX6231167.1 Bacteroidota bacterium
MDP1931898.1 Gammaproteobacteria bacterium
MEK7126161.1 Patescibacteria group bacterium
MBP7834939.1 Candidatus Saccharimonadota bacterium
MEK7554196.1 Patescibacteria group bacterium
WP 481701590.1 Spirosoma sp
phage 1044 gene1 PF13385
MCJ7583503.1 Anaerolineales bacterium
MCA9334549.1 Candidatus Saccharimonadota bacterium
MEI8231715.1 Actinomycetes bacterium
MFZ1301127.1 Candidatus Microsaccharimonas sp
WP 071540132.1 Polynucleobacter asymbioticus
MCK9602565.1 Sphaerochaeta sp
HRP30439.1 Agriterribacter sp
MCK9370928.1 Candidatus Dojkabacteria bacterium
CAL7961512.1 Gammaproteobacteria bacterium
MGD0020053.1 Candidatus Limnocylindrales bacterium
WP 439946504.1 Streptomyces sp BBFR109
WP 299588443.1 Mucilaginibacter sp
MCO5071152.1 Rhizobiaceae bacterium
WP 260763124.1 Candidatus Mycosynbacter amalyticus
MCA9360082.1 Candidatus Kaiserbacteria bacterium
KKR73256.1 Candidatus Nomurabacteria bacterium GW2011 GWA1 40 8
MCL5018881.1 Patescibacteria group bacterium
OGI28080.1 Candidatus Moranbacteria bacterium RIFOXYA1 FULL 44 7
WP 487542572.1 Devosia sp Soil C 22
HEX7769697.1 Dokdonella sp
HEY1044968.1 Bacteroidia bacterium
MHL5323644.1 Verrucomicrobiota bacterium
MDF2983148.1 Devosia sp
MFA5091694.1 Minisyncoccota bacterium
NLG04009.1 Clostridia bacterium
MDA3815064.1 Patescibacteria group bacterium
HOH62076.1 Bacilli bacterium
WP 393450843.1 Nocardia sp NPDC049149
WP 378720763.1 unclassified Nocardia
MDD4111362.1 Clostridia bacterium
WP 159540878.1 Aeromicrobium sp 9AM
WP 353028948.1 Mesorhizobium sp M1004
WP 459923258.1 Flavitalea antarctica
WP 095801783.1 Mesorhizobium sp WSM3866
WP 141564467.1 Mycolicibacterium palauense
MGV9002187.1 Candidatus Saccharimonadaceae bacterium
MBU1092540.1 Patescibacteria group bacterium
HEU4574616.1 Chitinophagaceae bacterium
MBI3654093.1 Acidobacteriota bacterium
phage 6441 gene1 PF13385
OHD23507.1 Spirochaetes bacterium GWB1 59 5
phage 3076 gene1 PF13385
MEJ2673302.1 Deltaproteobacteria bacterium
phage 1286 gene2 PF13385
phage 4760 gene1 PF13385
phage 3888 gene2 PF13385
phage 1277 gene1 PF13385
phage 849 gene1 PF13385
phage 3064 gene1 PF13385
phage 3082 gene1 PF13385
phage 907 gene2 PF13385
MHC4988733.1 Planctomycetota bacterium
MCP4145661.1 bacterium
WP 212005755.1 Chitinophaga sp HK235
WP 145671282.1 Chitinophaga polysaccharea
WP 218380167.1 Chitinophaga sp sic0106
WP 343668721.1 Chitinophaga sp
WP 146859916.1 Chitinophaga cymbidii
WP 095841127.1 Chitinophaga
MEO7535871.1 Ferruginibacter sp
MFT3679434.1 Ferruginibacter sp
WP 078351023.1 Mucilaginibacter pedocola
MES2875301.1 Bacteroidota bacterium
MFO0089307.1 bacterium
RYZ48846.1 Sphingobacteriales bacterium
RYF47887.1 Cytophagaceae bacterium
HRG02432.1 Bacteroidia bacterium
MBK6836723.1 Bacteroidota bacterium
MBP9688254.1 Bacteroidia bacterium
NDG54003.1 Flavobacteriia bacterium
WP 395652523.1 Brevundimonas sp
MFY8166728.1 Sediminibacterium sp
WP 293199337.1 Microcoleus sp PH2017 20 SFW D A
WP 293199384.1 Microcoleus sp PH2017 20 SFW D A
NBW38852.1 Cytophagia bacterium
NBU47933.1 Flavobacteriales bacterium
NBS75351.1 Betaproteobacteria bacterium
NBN87985.1 Candidatus Fonsibacter lacus
RLS85853.1 Planctomycetota bacterium
MHC5059358.1 Planctomycetota bacterium
phage 3116 gene1 PF13385
MCK4500826.1 Candidatus Babeliales bacterium
RZW57461.1 Flavobacteriaceae bacterium
MFY8170059.1 Candidatus Fonsibacter sp
MBV1952600.1 Cyclobacteriaceae bacterium
RYY51488.1 Chitinophagaceae bacterium
NBR98998.1 Actinomycetota bacterium
CAA6810403.1 uncultured Sulfurovum sp
NBV78200.1 bacterium
NBW35011.1 Cytophagia bacterium
NCX56316.1 Burkholderiaceae bacterium
CAK0766501.1 Gammaproteobacteria bacterium
KAB2916954.1 Bacteroidota bacterium
MEI6190509.1 Chitinophagia bacterium
MDG2285167.1 Alphaproteobacteria bacterium
MCS5550251.1 Gammaproteobacteria bacterium
MCR4307510.1 Candidatus Berkelbacteria bacterium
OHB92402.1 Planctomycetes bacterium RIFCSPHIGHO2 12 39 6
MEY2869133.1 Bacteroidota bacterium
MFN7583875.1 bacterium
HEX8517545.1 Bacteroidia bacterium
MCZ2337732.1 Chitinophagales bacterium
