# Supplementary material for: Bacteriophages in Antarctic subglacial lakes reveal novel biodiversity and biogeochemical potential
Source: ISME Commun. 2026 Sep 22;6(1):ycag252. doi: 10.1093/ismeco/ycag252 (PMC13615525; doi:10.1093/ismeco/ycag252)
Supplement: Supplementary file 1 [file supplementary_material_ycag252.zip › dcm_Supp4_ycag252.pdf]

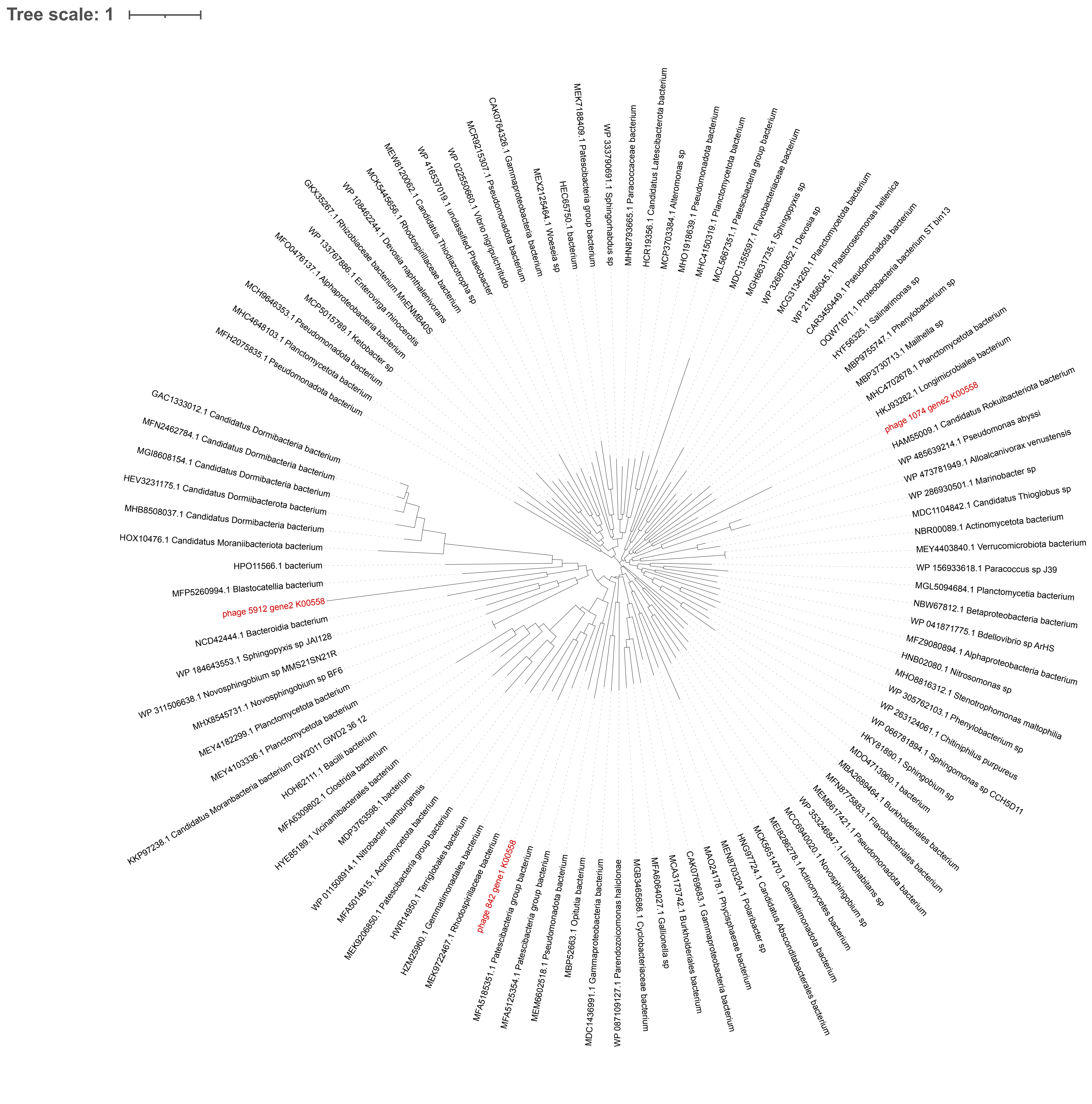

Tree scale: 1
GAC1333012.1 Candidatus Dormibacteria bacterium
MFN2462784.1 Candidatus Dormibacteria bacterium
MGI8608154.1 Candidatus Dormibacteria bacterium
HEV3231175.1 Candidatus Dormiibacterota bacterium
MHB8508037.1 Candidatus Dormibacteria bacterium
HOX10476.1 Candidatus Moraniibacteriota bacterium
HPO11566.1 bacterium
MFP5260994.1 Blastocatellia bacterium
phage 5912 gene2 K00558
NCD42444.1 Bacteroidia bacterium
WP 184643553.1 Sphingopyxis sp JAI128
WP 311506638.1 Novosphingobium sp MMS21SN21R
MHX8545731.1 Novosphingobium sp BF6
MEY4182299.1 Planctomycetota bacterium
MEY4103336.1 Planctomycetota bacterium
KKP97238.1 Candidatus Moranbacteria bacterium GW2011 GWD2 36 12
HOH62111.1 Bacilli bacterium
MFA6309802.1 Clostridia bacterium
HYE85189.1 Vicinamibacterales bacterium
MDP3763598.1 bacterium
WP 011508914.1 Nitrobacter hamburgensis
MFA5014815.1 Actinomycetota bacterium
MEK9206850.1 Patescibacteria group bacterium
HWR14950.1 Terriglobales bacterium
HZM25960.1 Gemmatimonadales bacterium
MEK9722467.1 Rhodospirillaceae bacterium
phage 842 gene1 K00558
MFA5185351.1 Patescibacteria group bacterium
MFA5125354.1 Patescibacteria group bacterium
MEM6602518.1 Pseudomonadota bacterium
MBP52663.1 Opitutia bacterium
MDC1436991.1 Gammaproteobacteria bacterium
WP 087109127.1 Parendozoicomonas haliclonae
MGB3465686.1 Cyclobacteriaceae bacterium
MFA6064027.1 Gallionella sp
MCA3173742.1 Burkholderiales bacterium
CAK0769683.1 Gammaproteobacteria bacterium
MAO24178.1 Phycisphaerae bacterium
MEN8703204.1 Polaribacter sp
HNG97724.1 Candidatus Absconditabacterales bacterium
MCK5651470.1 Gemmatimonadota bacterium
MEI8286278.1 Actinomycetes bacterium
MCC6940020.1 Novosphingobium sp
WP 353246847.1 Limnohabitans sp
MEM8617421.1 Pseudomonadota bacterium
MFN8775883.1 Flavobacteriales bacterium
MBA2689464.1 Burkholderiales bacterium
MDO4713960.1 bacterium
HKY81890.1 Sphingobium sp
WP 066781894.1 Sphingomonas sp CCH5D11
WP 263124061.1 Chitiniphilus purpureus
WP 305762103.1 Phenylobacterium sp
MHO8816312.1 Stenotrophomonas maltophilia
HNB02080.1 Nitrosomonas sp
MFZ9080894.1 Alphaproteobacteria bacterium
WP 041871775.1 Bdellovibrio sp ArHS
NBW67812.1 Betaproteobacteria bacterium
MGL5094684.1 Planctomycetia bacterium
WP 156933618.1 Paracoccus sp J39
MEY4403840.1 Verrucomicrobiota bacterium
NBR00089.1 Actinomycetota bacterium
MDC1104842.1 Candidatus Thioglobus sp
WP 286930501.1 Marinobacter sp
WP 473781949.1 Alloalcanivorax venustensis
WP 485639214.1 Pseudomonas abyssi
HAM55009.1 Candidatus Rokuibacteriota bacterium
phage 1074 gene2 K00558
HKJ93282.1 Longimicrobiales bacterium
MHC4702678.1 Planctomycetota bacterium
MBP3730713.1 Mailhella sp
MBP9755747.1 Phenylobacterium sp
HYF56325.1 Salinarimonas sp
OQW71671.1 Proteobacteria bacterium ST bin13
CAR3450449.1 Pseudomonadota bacterium
WP 211856045.1 Plastoroseomonas hellenica
MCG3134250.1 Planctomycetota bacterium
WP 326870852.1 Devosia sp
MGH6631735.1 Sphingopyxis sp
MDC1355597.1 Flavobacteriaceae bacterium
MCL5667351.1 Patescibacteria group bacterium
MHC4150319.1 Planctomycetota bacterium
MHO1918639.1 Pseudomonadota bacterium
MCP3703384.1 Alteromonas sp
HCR19356.1 Candidatus Latescibacterota bacterium
MHN8793665.1 Paracoccaceae bacterium
WP 333790691.1 Sphingorhabdus sp
MEK7188409.1 Patescibacteria group bacterium
HEC65750.1 bacterium
MEX2125464.1 Woeseia sp
CAK0764326.1 Gammaproteobacteria bacterium
MCR9215307.1 Pseudomonadota bacterium
WP 022550660.1 Vibrio nigripulchritudo
WP 416537019.1 unclassified Phaeobacter
MEW8120062.1 Candidatus Thiodiazotropha sp
MCK5445656.1 Rhodospirillaceae bacterium
WP 108462244.1 Devosia naphthalenivorans
GKX35267.1 Rhizobiaceae bacterium MnEMB40S
WP 133767886.1 Enterovirga rhinocerotis
MFO0476137.1 Alphaproteobacteria bacterium
MCP5015789.1 Ketobacter sp
MCH9646353.1 Pseudomonadota bacterium
MHC4648103.1 Planctomycetota bacterium
MFH2075835.1 Pseudomonadota bacterium
