# Supplementary material for: Bacteriophages in Antarctic subglacial lakes reveal novel biodiversity and biogeochemical potential
Source: ISME Commun. 2026 Sep 22;6(1):ycag252. doi: 10.1093/ismeco/ycag252 (PMC13615525; doi:10.1093/ismeco/ycag252)
Supplement: Supplementary file 1 [file supplementary_material_ycag252.zip › glycosyl_Supp3_ycag252.pdf]

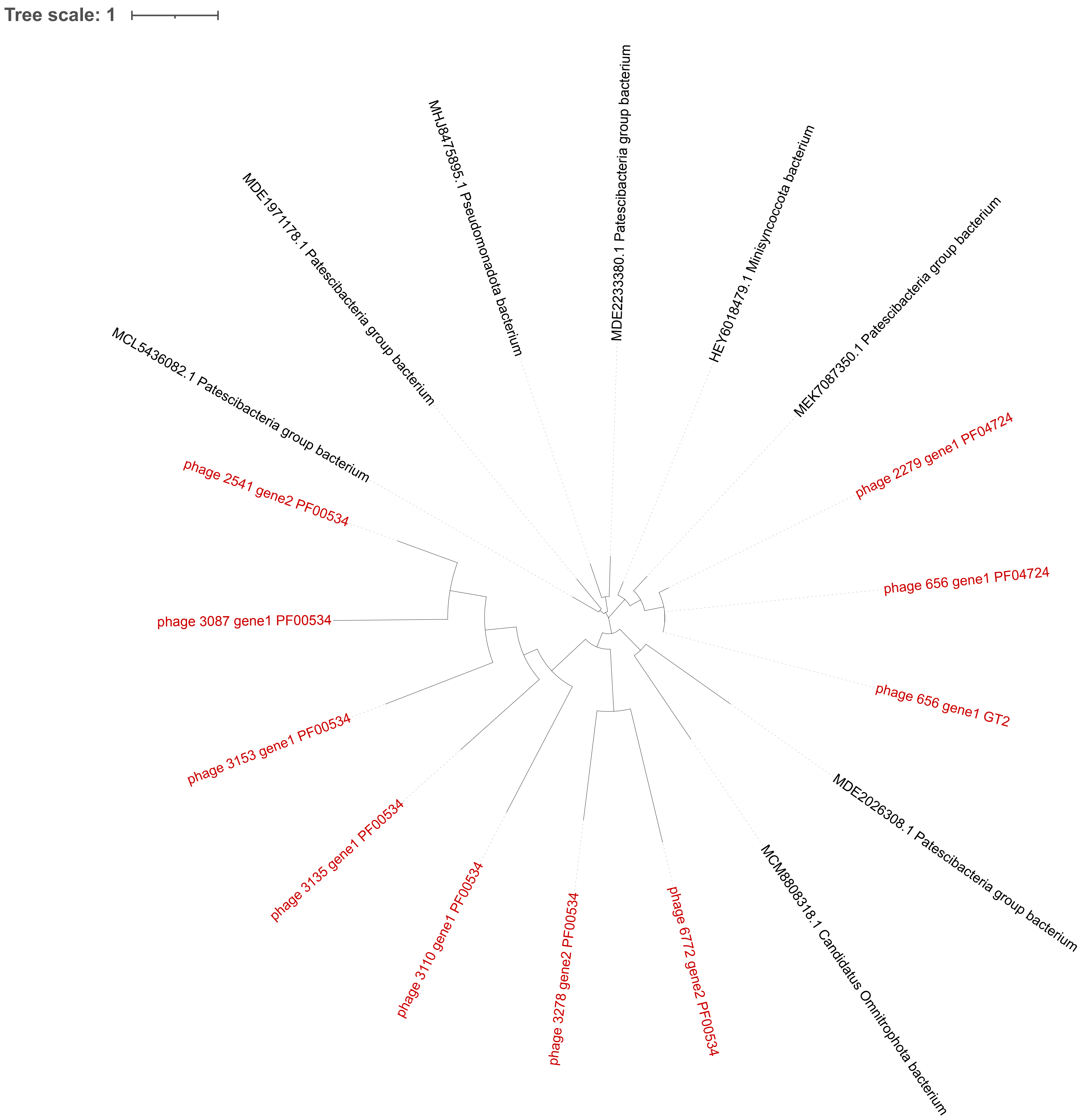
Tree scale: 1
MDE2233380.1 Patescibacteria group bacterium
HEY6018479.1 Minisyncoccota bacterium
MEK7087350.1 Patescibacteria group bacterium
phage 2279 gene1 PF04724
phage 656 gene1 PF04724
phage 656 gene1 GT2
MDE2026308.1 Patescibacteria group bacterium
MCM8808318.1 Candidatus Omnitrophota bacterium
phage 6772 gene2 PF00534
phage 3278 gene2 PF00534
phage 3110 gene1 PF00534
phage 3135 gene1 PF00534
phage 3153 gene1 PF00534
phage 3087 gene1 PF00534
phage 2541 gene2 PF00534
MCL5436082.1 Patescibacteria group bacterium
MDE1971178.1 Patescibacteria group bacterium
MHJ8475895.1 Pseudomonadota bacterium
