# Supplementary material for: Bacteriophages in Antarctic subglacial lakes reveal novel biodiversity and biogeochemical potential
Source: ISME Commun. 2026 Sep 22;6(1):ycag252. doi: 10.1093/ismeco/ycag252 (PMC13615525; doi:10.1093/ismeco/ycag252)
Supplement: Supplementary file 1 [file supplementary_material_ycag252.zip › SeqMetrics_Supp1_ycag252.pdf]

Lake	Sample	Format	Type	Num_Seq	Sum_Len
Mercer	SLM_CELL_1	FASTQ	DNA	40,389,308	6,049,303,314
Mercer	SLM_CELL_2	FASTQ	DNA	40,389,308	6,042,239,618
Mercer	SLM_VIRAL_1	FASTQ	DNA	40,200,936	6,008,297,849
Mercer	SLM_VIRAL_2	FASTQ	DNA	40,200,936	6,000,784,742
Whillans	SLW_1	FASTQ	DNA	3,936,778	382,869,648
Whillans	SLW_2	FASTQ	DNA	3,936,778	383,947,390

Supplemental Table 1: Sequencing metrics of the quality-controlled reads. SLM_CELL = SLM cellular fraction; SLM_VIRAL = SLM dedicated viral fraction; SLW = SLW cellular fraction. Numbers following sample names (1, 2) indicate paired-end read files (forward and reverse reads). Columns show the total number of reads per file and the total number of bases sequenced.
